# From Radiomics to Radiogenomics: Decoding Renal Cell Carcinoma Biology for Precision Medicine—a narrative review

**DOI:** 10.1186/s13244-025-02164-6

**Published:** 2025-12-17

**Authors:** Zihan He, Liping Huang

**Affiliations:** Department of Ultrasound, Shengjing Hospital of China Medical University, Shenyang, Liaoning China

**Keywords:** Renal cell carcinoma, Radiomics, Radiogenomics, Precision medicine, Artificial intelligence

## Abstract

**Abstract:**

Renal cell carcinoma is a prevalent malignancy affecting the urinary system and poses significant challenges in precision diagnosis and treatment. Although medical imaging technologies have been widely applied in renal cell carcinoma screening, traditional imaging diagnostics have limitations due to their high degree of subjectivity, relying primarily on the doctor’s experiential judgment. The advent of radiomics presents a groundbreaking method for tackling this issue—by extracting high-throughput, deep-level information from conventional medical images to achieve a quantitative assessment of tumor characteristics. Furthermore, the fusion of radiomics and genomics has led to radiogenomics, which combines imaging features with molecular data, enabling the non-invasive evaluation of tumor biological behavior, molecular heterogeneity, and microenvironmental features, thereby providing a more detailed, accurate, and personalized assessment. In this review, we summarize the role radiomics and radiogenomics play in the diagnosis, prediction, and adjuvant treatment of renal cell carcinoma. Radiomics has demonstrated potential in classifying renal cell carcinoma subtypes, predicting patient prognosis, and forecasting disease progression. Radiogenomics further links imaging features to gene mutations and the tumor microenvironment, enabling non-invasive assessment of renal cell carcinoma biology and providing new approaches to diagnosis and treatment.

**Critical relevance statement:**

By reviewing existing research, we summarize how radiomics and radiogenomics address key clinical challenges in the diagnosis and treatment of renal cell carcinoma, providing non-invasive solutions to overcome tumor heterogeneity and guide precision oncology.

**Key Points:**

- Renal cell carcinoma lacks reliable non-invasive biomarkers for precision diagnosis and characterization.
- Radiogenomics bridges imaging and molecular biology for precise predictions.
- Radiogenomics lacks full multi-omics integration despite data growth.

**Graphical Abstract:**

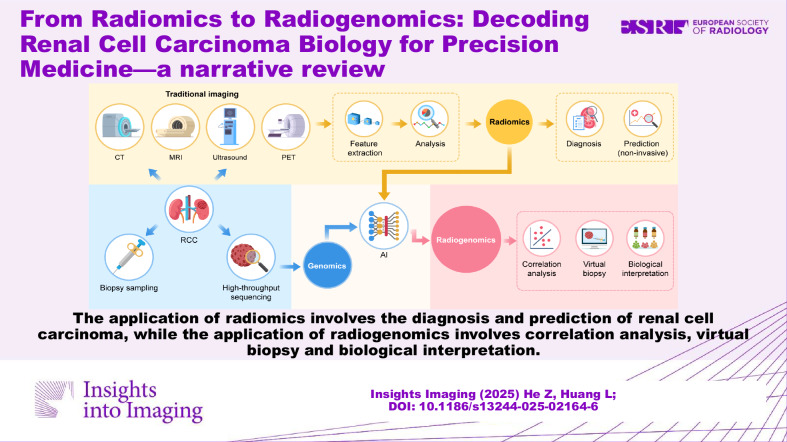

## Introduction

Renal cell carcinoma (RCC) ranks as the 14th most common malignant tumor globally and is the third most prevalent urological malignancy in men, following prostate and bladder cancers. It accounts for 3%–5% of all malignant tumors, with roughly 430,000 new diagnoses and 150,000 fatalities reported globally in 2022. The incidence is particularly high in Europe and North America, posing a considerable hazard to public health [1, 2]. According to the World Health Organization (WHO) Classification of Malignant Tumors of the Male Genital Organs 2022, RCC can be classified into at least six different histologic subtypes, with clear cell RCC (ccRCC), papillary RCC (pRCC), and chromophobe RCC (chRCC) being the most common, and rare subtypes include collecting duct RCC and medullary RCC [3]. Owing to the high molecular heterogeneity of RCC and its complex tumor microenvironment (TME), conventional imaging cannot accurately assess its biological characteristics, restricting personalized therapeutic approaches.

Imaging is crucial for diagnosing and treating RCC. Medical imaging modalities like ultrasound, CT, and MRI are commonly employed in the diagnosis and evaluation of kidney tumors because they are non-invasive and readily available in clinical settings. However, traditional imaging heavily relies on the subjective judgment of doctors, which can lead to bias. In up to 20–30% of cases, imaging results in false-positive diagnoses of malignant lesions [4–6]. Furthermore, imaging fails to reflect the heterogeneity within tumors. With advances in high-throughput genomics, the paradigm of cancer treatment has undergone significant changes [7, 8]. However, genomics relies on tumor samples obtained through invasive means, such as surgery or biopsy, an approach that has the disadvantage of reflecting only local tumor characteristics and is not suitable for dynamic monitoring. Radiomics has become a rapidly growing area of research in recent years. It has seen extensive use, particularly in cancer studies, by efficiently extracting numerous features from medical images, quantifying these features, and exploring how they relate to disease progression and patient outcomes. Radiomics has the advantage of completely and noninvasively characterizing the full landscape of a tumor. However, owing to the high molecular heterogeneity and complex TME, radiomics cannot accurately assess its biological characteristics, restricting tailored therapeutic approaches. Radiogenomics is a new and more comprehensive technology that aims to complement the strengths of both genomics and radiomics, as well as to serve as a bridge between radiomic and microscopic features of disease, further elucidating the biological interpretation of radiomic features. This would provide more relevant information and improve the accuracy of prediction [9].

Unlike previous studies [10–12], this review summarizes the latest literature, focusing on the diagnostic, predictive, and therapeutic applications of radiomics and radiogenomics in RCC, while also covering the genetic and microenvironmental basis of radiogenomics in RCC. It connects the key drivers of RCC with radiogenomics biomarkers. Additionally, we analyzed the current challenges and proposed targeted solutions.

## Key drivers in renal cell carcinoma: molecular and microenvironmental bases for radiogenomics

### Gene mutations

With the deepening of genomic research, gene mutations were discovered to be pivotal in the progression of RCC, both by directly affecting cell proliferation, metabolism, and death, and by disrupting critical signaling pathways that, in turn, promote drug-resistant phenomena, such as tumor invasion and cellular resistance to death. A systematic analysis identified 24 high-frequency mutated genes strongly linked to RCC development, progression, and therapy. After comparing their distribution across different RCC subtypes, *VHL* gene mutations were found to occur significantly more frequently in ccRCC than in pRCC and chRCC [13]. A genome-wide association study indicated that *VHL* loss is a key driver of ccRCC development [14]. In contrast, the core drivers of pRCC vary by tumor grade: low-grade pRCC exhibits *MET* proto-oncogene mutations, whereas high-grade pRCC frequently involves NRF2-ARE pathway activation and *CDKN2A/TP53* alterations [15]. The key driving event in chRCC involves rearrangement of the *TERT* promoter [16]. Other gene mutations are also involved in the formation of RCC: *PBRM1* is potentially associated with tumor immune escape; *BAP1 and SETD2* participate in DNA repair regulation; and *MTOR* regulates the critical PI3K/AKT/mTOR signaling pathway [17–19]. Studies such as these provide key insights into the pathogenesis of RCC but also furnish online databases for use in more detailed radiogenomics analyses.

### Signaling pathways

The *VHL*-HIF axis is the most important aberrant signaling pathway in RCC [20]. Inactivation of *VHL* impairs HIF-α degradation, allowing it to enter the cell nucleus and bind to the hypoxia-inducible element. This activates downstream targets, such as VEGF and PDGF, in tumor cells, driving tumor angiogenesis [21]. Drugs targeting the *VHL*-HIF pathway (such as sunitinib and bevacizumab) have significantly prolonged survival in patients with RCC by inhibiting angiogenesis and other mechanisms [22].

The PI3K/AKT/mTOR pathway also plays a critical role in RCC. PIK3CA mutations promote the conversion of PIP2 to PIP3 [23], whereas PTEN mutations result in the loss of its ability to dephosphorylate PIP3, ultimately activating the AKT/mTOR pathway [24]. As a core downstream effector, mTOR regulates tumor growth and survival through the mTORC1/2 complex [25] and is a key therapeutic target for RCC, with mTOR inhibitors (such as everolimus) already in clinical use. In recent years, novel dual PI3K/mTOR inhibitors have shown promise for more comprehensive signaling blockade, though their safety profiles require further investigation [26].

### Tumor microenvironment

The resistance of RCC to conventional therapies is not only affected by genetic mutations but also significantly by the TME, a complex system composed mainly of cancer cells, immune cells, fibroblasts, endothelial cells, and extracellular matrix (ECM). Therefore, a better understanding of the TME may help us to develop new therapies for RCC treatment.

#### High angiogenic features

RCC is a vascular-rich tumor [27], mainly due to inactivation of the mutant gene *VHL*, leading to an abnormal accumulation of HIF-1/2α and initiating an angiogenic program with elevated levels of VEGF. Past treatments for metastatic RCC (mRCC) have primarily targeted the VEGF signaling pathway, but some patients have developed drug resistance. The combination therapy of pembrolizumab, an immune checkpoint inhibitor, and axitinib, a VEGF receptor tyrosine kinase inhibitor (TKI), demonstrated marked improvements in survival outcomes for patients with RCC. This dual approach delivered superior clinical outcomes compared to those of conventional treatments [28].

#### Immune infiltrate heterogeneity

RCC ranks among the solid tumors with the most extensive immune cell infiltration, which is a key factor in immunotherapy resistance. Within the TME, T cells (primarily CD4⁺ and CD8⁺ subsets) represent the main immune cell populations [29]. Despite abundant infiltration of CD8⁺ T cells as primary anti-tumor effectors, their function is compromised due to immunosuppression. Tumor-associated macrophages (TAMs), regulatory T cells (Tregs), and myeloid-derived suppressor cells (MDSCs) inhibit CD8⁺ T-cell functionality through PD-1, CTLA-4, and TIM-3 receptor activation, causing T-cell exhaustion and functional impairment [30]. Rapid tumor proliferation leads to a hypoxic microenvironment. Research indicates that patients with high hypoxia-risk scores develop an immunosuppressive tumor environment, marked by significantly elevated immunosuppressive cells (such as Tregs) and concurrently suppressed efficient immune effector cells (such as monocytes and M1 macrophages), ultimately promoting tumor immune escape [31].

#### Extracellular matrix remodeling

**Fig. 1:**
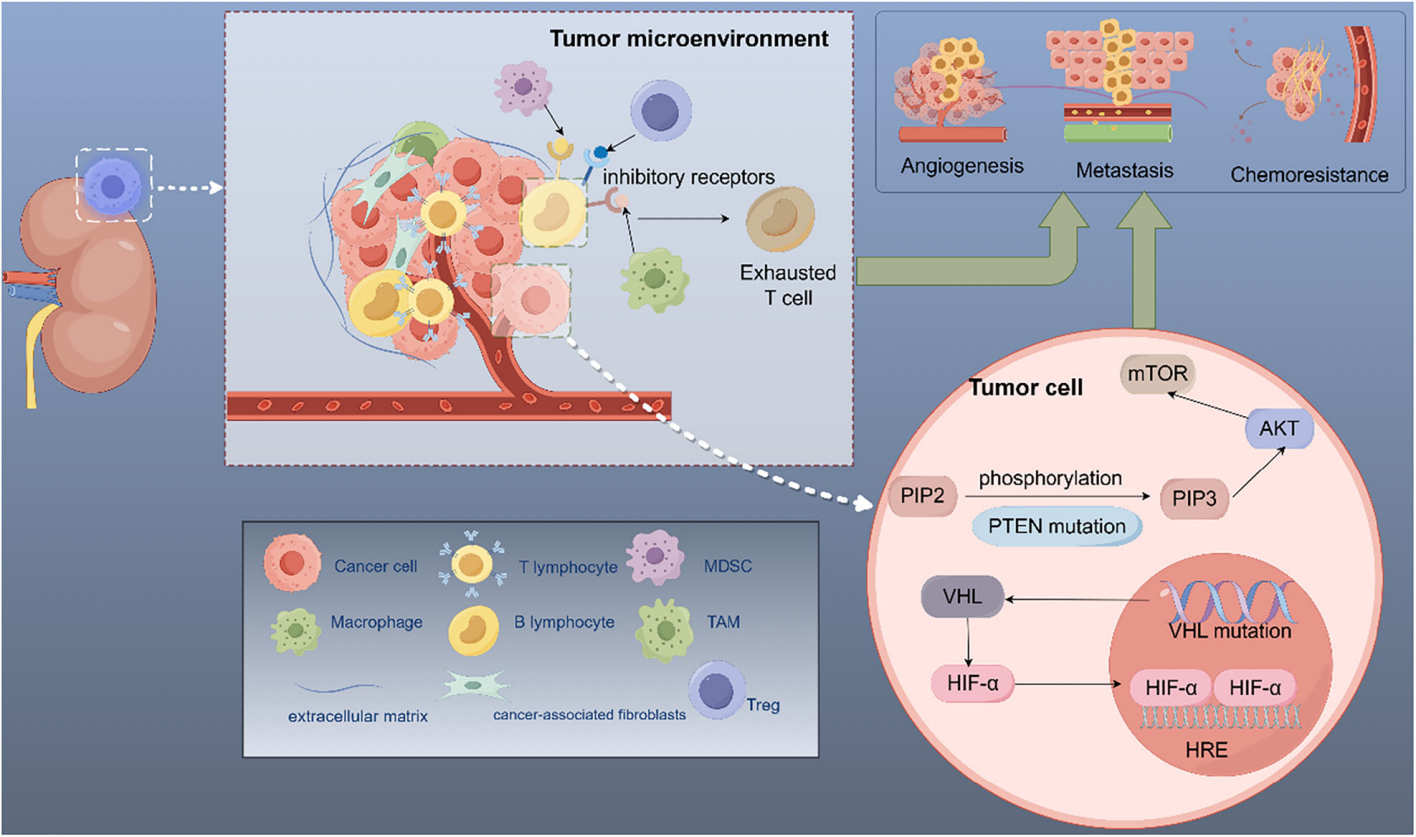
Key signaling pathways and tumor microenvironment regulation involved in the pathogenesis of renal cell carcinoma. The key molecular drivers and tumor microenvironment characteristics of renal cell carcinoma collectively contribute to adverse outcomes such as tumor metastasis, angiogenesis, and chemoresistance

Cancer-associated fibroblasts (CAFs) are key components of the TME. Under physiological conditions, fibroblasts act as a major component of the ECM of connective tissues, maintaining ECM homeostasis and initiating repair functions in case of tissue damage [32]. Fibroblasts critically influence cancer progression. These cells undergo permanent activation in pathological states and transform into CAFs, which serve as key stromal components of the TME. CAFs promote cancer cell migration and metastasis by remodeling connective tissues and secrete a variety of factors that drive tumor progression, angiogenesis, and therapeutic resistance [33] (Fig. 1).

## Application of radiomics in renal cell carcinoma

A summarized version of the articles mentioned in the subsequent sections can be found in Table 1.

**Table 1:** Summary of selected recent studies in radiomics for renal cell carcinoma

Study	Task	lmaging	Key radiomics features	Model	Performance (best model)/findings
[35]	Classification	CT	Texture	ANN and SVM	Internal: MCC: 0.834 for ccRCC vs. non-ccRCC External: MCC: 0.728 for ccRCC vs. non-ccRCC
[36]	Classification	MRI	Texture	LR and LDA	Internal: AUC: 0.959 for ccRCC vs. chRCC AUC: 0.955 for pRCC vs. chRCC AUC: 0.890 for ccRCC vs. pRCC
[37]	Classification	CT	Not specified	DenseAUXNet201 and XGBoost	Internal: Accuracy: 85.66%, Precision: 84.18% Recall: 85.66%, F1-score: 84.92%
[39]	Treatment response	CT	First-order, texture	SVM	Internal: Pre-treatment AUC: 0.46 Post-treatment AUC: 0.51
[40]	Treatment response	CT	First-order	Mann–Whitney test and Pearson correlation coefficient	Associations between several radiomics features and immunotherapy efficacy in advanced RCC
[46]	Metastasis	CT	Shape, first-order, and texture	SVM, RF, LR, GP, and Bagging DT	Internal: AUC: 0.935, 95% CI [0.902–0.968] External: AUC: 0.891, 95% CI [0.794–0.988]
[47]	Metastasis	CT	Texture and wavelet	*k*-means clustering, RF, and mi-SVM	Internal: AUC: 0.805
[52]	Recurrence	CT	Wavelet	Ridge regression, homogenous ensemble, and LASSO	Internal: C-index: 0.80, 95% CI [0.76–0.86]
[53]	Recurrence	CT	Texture	Cox regression, affinity propagation clustering, and RSF	Internal: iAUC: 0.78 95% CI [0.69–0.88]
[56]	Overall survival	CT	Shape, first-order, texture, and wavelet	LASSO Cox regression	Internal: C-index: 0.884, 95% CI [0.80–0.940] External: C-index: 0.859, 95% CI [0.80–0.921]
[57]	Disease-free survival	CT	Shape, first-order, and wavelet	LASSO Cox regression	Internal: AUC: 0.781, 95% CI [0.728–0.833] External: AUC: 0.754, 95% CI [0.689–0.821]

*ANN* artificial neural network, *BaggingDT* bagging decision tree, *GP* Gaussian process, *iAUC* incremental area under the curve, *LASSO* least absolute shrinkage and selection operator, *LDA* linear discriminant analysis, *LR* linear regression, *MCC* Matthews correlation coefficient, *mi-SVM* multi-instance support vector machine, *RF* Random Forest, *RSF* random survival forest, *SVM* support vector machine

### Radiomics-based diagnosis of renal cell carcinoma

RCC comprises several histological subtypes. Given the broad use of precision treatments and immunotherapy, treatment efficacy varies substantially across subtypes. Accurate RCC classification would thus enable optimized therapeutic strategies [34]. Kocak et al applied quantitative CT texture analysiscombined with machine learning classifiers to corticomedullary phase images, developing a model that distinguished ccRCC from non-ccRCC with 84.6% accuracy upon external validation. However, its performance in differentiating the three major RCC subtypes remained suboptimal [35]. For other imaging modalities, Wang et al identified significant radiomics feature differences among ccRCC, pRCC, and chRCC across three MRI sequences (T2WI, EN-T1WI CMP, and EN-T1WI NP). Linear discriminant analysis demonstrated a combined sequence accuracy rate of 71.4% [36]. Mahmud et al pioneered a machine learning and deep learning model that integrated CT imaging and clinical metadata, achieving 85.66% accuracy in RCC subtype classification. Multimodal data significantly enhanced its performance. Feature analysis highlighted tumor size and cancer phase as pivotal determinants for surgical decision-making, and the integration of clinical data substantially improved model efficacy [37]. Overall, radiomics can extract radiomic features through machine learning to reflect the imaging features of different RCC subtypes. However, the advantages of different imaging modalities and their enhancement patterns, as well as their integration with clinical parameters, require further evaluation.

Currently, studies related to predicting RCC subtypes have focused on ccRCC, chRCC, and pRCC. Significant differences in the malignancy and drug sensitivity of other renal cancer subtypes exist; therefore, identifying different subtypes of RCC requires further investigation.

### Radiomics-based prediction of renal cell carcinoma

#### Treatment response

Against the backdrop of immune checkpoint inhibitors serving as a common treatment for advanced RCC, evaluating treatment efficacy solely through changes in tumor volume is incomplete [38]. Radiomics has garnered attention as a promising biomarker for predicting treatment response. To predict responders and non-responders to nivolumab, Malone et al developed a predictive model using machine learning-based radiomics analysis of pre- and post-treatment CT scans. The model, however, demonstrated poor discriminatory ability and lacked significant predictive value [39]. Rossi et al analyzed the association between radiomic features extracted from patients with advanced RCC and immunotherapy response, finding that higher values of certain highly dense and heterogeneous features (F_stat.var and F_stat.max) within specific tumor regions could correlate with increased progression risk [40]. The predictive value of radiomic features for immunotherapy response requires further validation through additional studies. Future research should integrate deep learning with large, multicenter datasets to optimize patient stratification and reduce the risk of treatment failure.

Immunotherapy has been widely employed for mRCC treatment. Further prediction of their therapeutic efficacy will be crucial to improve patient survival time and reduce treatment-related burden. Future deep learning models should predict the effectiveness of immune therapies.

#### Metastasis

Currently, ccRCC represents the most common and clinically aggressive RCC, and distant metastasis occurs in 18%–30% of patients upon initial diagnosis [41, 42]. Research indicates that those with ccRCC and subsequent metastasis face unfavorable outcomes [43]. Traditional radiomics is analyzed on the region of the entire tumor, and this approach defaults to the idea that tumor heterogeneity is uniformly and randomly distributed throughout the tumor; however, this is not the case. Thus, the traditional whole-tumor-based radiomics model fails to adequately reflect tumor heterogeneity [44]. Recent studies have shown that Habitat imaging, which divides tumors into subregions with similar characteristics called habitats, can better reflect tumor heterogeneity [45]. A multicenter study classified ccRCC tumors into different habitats based on multiphase enhanced CT images and constructed a multimodal prediction model integrating subregion and volume characteristics, as well as ultrasound and clinical information, and found that specific subregions (especially Habitat3), which exhibited medium-high density with heterogeneous enhancement zones, were closely associated with postoperative metastasis. Distant metastasis is closely related to the CT_Habitat3 [46]. In another study, a multi-instance learning framework was introduced to classify each tumor into three subregions and identify the “high-risk subregions” that are most likely to drive synchronous distant metastasis by using the mi-SVM model. Subregion 2, which is located at the peripheral zone around the tumor and transition zone between the solid and liquid components within the tumor, was identified as the key region [47]. Future large-scale prospective studies can further validate the reliability of the model and predict the metastatic sites in ccRCC.

Although the above studies used different algorithms and data sources, they all demonstrated the predictive significance of regions of heterogeneity within tumors, providing data support for preoperative risk stratification of ccRCC, individualized treatment, and follow-up.

#### Recurrence

Approximately 20% of localized RCC cases recur postoperatively [48]. The KEYNOTE-564 study demonstrated that adjuvant pembrolizumab improves survival in high-risk patients [49, 50]. However, widespread use will increase the risk of adverse events [51]. Therefore, this study aims to accurately assess recurrence risk to enhance patient selection for beneficial adjuvant immunotherapy. A retrospective study analyzed 453 patients with non-mRCC and compared the performance of the clinical model with that of the combined model by integrating postoperative clinical-pathological indices with preoperative CT radiomic features through Cox modeling. The findings demonstrated that integrating CT radiomic features enhances the predictive accuracy of recurrence risk in non-mRCC surgical patients within the prognostic model [52]. Another multicenter study further developed a risk stratification model integrating radiomics and clinical information, demonstrating superior performance to traditional staging systems in high-risk patients. This model has been clinically translated through an online platform [53]. However, the current bottleneck of this technology is that image segmentation still requires manual intervention. In the future, more models (such as deep learning) that can be integrated into the entire predictive analytics workflow will need to be developed.

Due to the pronounced heterogeneity of RCC, patient outcomes vary considerably. The above study demonstrated that radiomics improves the assessment of postoperative recurrence risk in patients with RCC and helps guide adjuvant treatment decisions.

#### Survival ending

The conventional method for predicting RCC outcomes mainly depends on the tumor-node-metastasis (TNM) classification by the American Joint Committee on Cancer [54] and histopathological nuclear grade [55]. Due to the clinical heterogeneity of tumors, clinical outcomes may vary significantly, even among patients in the same category with the same stage. Radiomics forecasts patient outcomes in RCC by predicting overall survival (OS) and disease-free survival (DFS). Yan et al constructed a predictive model by integrating the Rad-score with clinical indicators such as TNM staging, demonstrating significantly higher accuracy than models relying solely on clinical indicators. This confirms that radiomics provides incremental value for prognostic assessment [56]. Xu developed a deep learning prognostic model for patients with localized ccRCC, successfully achieving risk stratification for DFS. The model demonstrated outstanding performance in external validation (C-index: 0.754), outperforming conventional models such as the UISS and Leibovich scores [57]. In summary, radiomics-based prognostic scoring systems have demonstrated efficacy in predicting survival rates for ccRCC. However, the potential of deep learning radiomics in assessing OS for advanced and metastatic ccRCC warrants further investigation.

**Fig. 2:**
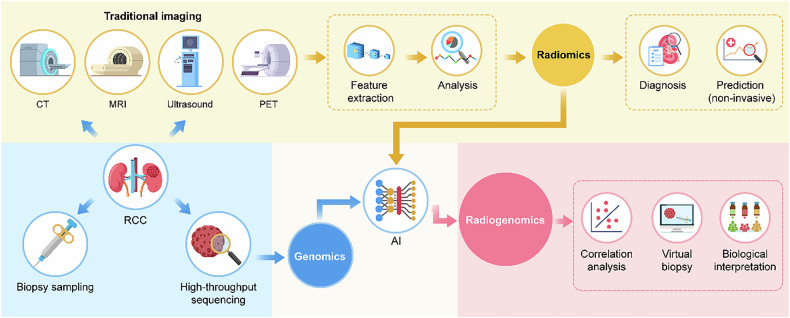
Applications and processes of radiomics and radiogenomics in renal cell carcinoma. The schematic diagram illustrates the process of integrating radiomics and genomics into radiogenomics for the precision diagnosis and treatment of renal cell carcinoma

In contrast with the poorest 5-year survival rate exhibited by ccRCC, pRCC and chRCC are less aggressive [58], and the majority of studies predicting survival outcomes have thus focused on ccRCC. Preoperative risk assessment of advanced-stage patients with a poorer prognosis facilitates treatment decisions. For patients with poor survival prognosis, treatment strategies can be adjusted in a timely manner by considering the use of targeted therapy and immunotherapy as early as possible. (Fig. 2).

## Application of radiogenomics in renal cell carcinoma

A summarized version of the articles mentioned in the subsequent sections can be found in Table 2.

**Table 2:** Summary of selected recent studies in radiogenomics for renal cell carcinoma

Study	Biological feature	Imaging feature	Integration method	Key findings/correlation
[63]	*VHL*, *PBRM1*, and *KDM5C* mutations	Tumor margins, enhancement pattern, renal vein invasion	Fisher’s exact test, independent *t*-test	Associations between CT features and underlying mutations
[65]	mRNA-based subtypes	Tumor margins, renal vein invasion, and collecting system invasion	Logistic regression	Associations between CT features and mRNA-based subtyping
[66]	microRNA expression	CTTA parameters	*r* and *R*^2^	Associations between CTTA and microRNAs
[69]	259-gene prognostic signature	Tumor necrosis, tumor transition zone, tumor-parenchyma interaction, tumor-parenchyma interface	Linear regression	Predict disease-specific survival
[70]	*VHL*, *PBRM1*, and *BAP1* mutations	Geometry, intensity, texture features	MCMO model	Predict specific gene mutations
[73]	*RUNX3* methylation	Tumor margin, side, long diameter, and intratumoral vascularity	Logistic regression	Predict high *RUNX3* methylation
[74]	DNA methylation subtypes	Tumor size, necrosis, and enhancement pattern	Logistic regression	Predict DNA methylation subtypes and overall survival rates
[78]	Hypoxia-related gene signature	Thirteen optimal radiomics features	mRMR, LASSO, logistic regression	Predict hypoxia-gene signature expression levels and prognosis in patients
[82]	Molecular pathways, TME	Texture, intensity, and semantic features	r	Predictive of metastasis risk and reflective of underlying tumor biology
[83]	Tumor angiogenesis, cell adhesion, and extracellular structure organization	Texture and shape features	WGCNA, RF	Predict prognosis and reveal the underlying molecular pathways
[84]	T-cell activation	Ten optimal radiomics features	LASSO, multivariate Cox analysis, WGCNA	Predict PFI and reveal the underlying immune-system cell response
[85]	ECM-receptor interaction, focal adhesion, PI3K-Akt pathways	Texture features	LASSO, WGCNA	Predict metastasis and reveal the underlying biological pathways

*CT* computed tomography, *CTTA* computed tomography texture analysis, *ECM* extracellular matrix, *LASSO* least absolute shrinkage and selection operator, *MCMO* multi-classifier multi-objective, *mRMR* minimum redundancy maximum relevance, *PFI* progression-free interval, *r* Pearson correlation coefficient, *R*^2^ coefficient of determination, *RF* Random Forest, *TME* tumor microenvironment, *WGCNA* weighted gene correlation network analysis

### Correlation analysis

With the advancement of genetic testing technology, targeted therapy and immunotherapy have transformed conventional treatments. In recent years, radiogenomics, as a non-invasive testing technology, has opened a new direction for precision tumor treatment by exploring connections between imaging features and genetic characteristics [59–62]. Karlo et al initially examined associations between imaging features and gene mutations in ccRCC. The analysis of CT images of 233 patients with ccRCC revealed that the *VHL* gene mutation was significantly associated with the features of tumors with clear margins and abundant internal blood vessels, whereas the *BAP1* and *KDM5C* mutations were associated with tumor invasion into the renal vein, which tentatively suggests that different gene mutations may be reflected through specific imaging features [63].

Radiogenomics not only responds to gene mutations but also to abnormalities at the levels of gene expression (mRNA level) and regulation upstream (miRNA level). Traditional pathological classification currently struggles to meet the demands of personalized treatment, making gene expression profiling a focal point of research. The Cancer Genome Atlas (TCGA) studies have classified ccRCC into four mRNA subtypes (M1–M4), and different subtypes have different biological characteristics and clinical prognoses [14, 64]. Lan was the first to study the relationship between CT imaging features and mRNA subtypes in ccRCC. The study results showed that the M1 subtype was more likely to have a well-defined margin, whereas the M3 subtype was more likely to have an ill-defined margin and invade the renal vein [65]. Furthermore, Marigliano et al. found that CT texture parameters (entropy values) showed a strong positive correlation with miRNA expression, including miR-21-5p [66].

Correlation analysis has preliminarily established correlations between imaging features and multi-level molecular characteristics, laying the foundation for non-invasive identification of molecular subtypes and personalized treatment decisions through radiogenomics.

### Virtual biopsy

As treatment continues to advance, molecular heterogeneity changes at different sites within the bodies of patients with cancer, and traditional biopsies are unable to repetitively sample tissue from multiple sites to accurately characterize tumor heterogeneity [67]. Compared with the previous exploratory correlation analysis, virtual biopsy (VB) focuses on model construction and clinical translation. Its core goals include replacing invasive biopsies, optimizing treatment decisions, and facilitating real-time dynamic monitoring. Jamshidi et al combined a 259-gene supervised principal component risk score [68] with four CT imaging features to construct a radiogenomic risk score. Results demonstrated that this score exhibits high accuracy, reveals associations with survival rates, and independently predicts patient survival beyond clinical staging [69]. Ensemble learning algorithms also show promising prospects. Chen et al integrated six distinct classifiers to develop a multi-classifier multi-objective radiogenomics model. Results demonstrated that this ensemble model achieved AUC values > 0.85 for predicting mutations in VHL, PBRM1, and BAP1 genes, significantly outperforming individual classifiers [70].

DNA methylation serves as a diagnostic and prognostic biomarker [71, 72]. A study found that patients with high *RUNX3* methylation exhibited shorter survival times, and specific CT features (e.g., intratumoral vascularity) effectively predicted methylation status. This could provide a non-invasive prognostic assessment tool for patients with ccRCC and inform personalized therapy. However, the study’s sample was confined, necessitating additional verification with a more extensive data pool [73]. Yu et al further analyzed the TCGA/TCIA database and found that RCC subtypes (M1–M3) based on DNA methylation exhibit distinct CT imaging phenotypes: M1 (worst prognosis) typically presents with a long axis ≥ 70 mm and necrosis; M2 often shows nodular enhancement without necrosis; M3 is frequently smaller in size with necrosis. This suggests that CT features can serve as imaging biomarkers for non-invasive classification [74].

At the level of gene expression, radiogenomics reflects the “pseudohypoxia” metabolic state characteristic of ccRCC [75–77]. Gao et al developed the Rad-score based on hypoxia-regulated genes, comprising 13 key imaging features that effectively distinguish high- and low-risk groups for hypoxia-gene expression. This demonstrates a quantitative correlation between imaging phenotypes and HIF pathway-driven metabolic reprogramming, offering a novel approach for non-invasive assessment of the TME [78].

VB aims to support precision medicine by building predictive models through imaging genomics and machine learning to achieve the non-invasive prediction of gene mutations, molecular subtypes, or characteristics of the TME, thus realizing a “virtual biopsy”.

### Biological interpretation

The ultimate goal of radiogenomics extends beyond prediction or classification; more importantly, it aims to distinguish patient subgroups that will benefit from personalized therapies (including targeted therapies) by leveraging the underlying histological and molecular characteristics behind imaging findings [62]. Despite being mostly stage T1 and surgically resectable at the time of detection [79], approximately 30% of patients may still develop distant metastases after surgery [80]. Traditional TNM staging systems do not fully reflect the risk of postoperative metastasis [79–81]. Lee et al pioneered the development of a radiomics model based on CT imaging features for predicting postoperative metastasis in patients with pathologic stage T1 RCC. By correlating the imaging features with gene expression using RNA-seq and validation and biopathway enrichment analysis using TCGA-KIRC data, the gene sets associated with each image feature were found to be enriched in specific pathways such as PI3K/AKT and Wnt signaling pathways. An extensive evaluation also examined correlations among radiomic characteristics, immune cell populations, and immune checkpoint molecule expressions, and the findings revealed elevated immunosuppressive cell activity with immune escape features in the high-risk cohort. Moreover, specific imaging features were significantly associated with the expression of immune checkpoints, such as PD-1, LAG3, and CTLA4 [82]. To predict the OS of patients with ccRCC, Huang et al extracted 107 imaging genomic features from 205 patients and found that the green module was highly correlated with prognostic-related radiographic features. Finally, an RF algorithm was applied to integrate the imaging features and gene co-expression modules, and an image-genomics fusion factor was proposed. To further elaborate on the potential molecular pathways associated with the prognostically important green gene module, an enrichment analysis was performed in this study, and the results indicated that the module genes might be involved in tumor angiogenesis, cell adhesion, and extracellular structure organization [83]. Gao et al predicted the progression-free survival of patients with ccRCC and established significant correlations between radiological features and aspects of T-cell activation, immune synapses, and chemokine activity, suggesting that radiological profiles can serve as a non-invasive predictor of immuno-oncology profiles and may be helpful in the treatment and prognostic management of patients with cancer [84]. Zhao et al constructed a metastasis score based on CT imaging histology for predicting the ability of localized ccRCC to metastasize distantly and revealed key biological pathways associated with predictive radiomic features. Unlike in previous studies, a radiomics-transcriptomics-metastasis co-analysis approach was used to predict the biological basis of the radiomic signature of ccRCC metastasis, rather than merely building a radiomic model that predicts the outcome. WGCNA was also used to identify four significantly enriched pathways associated with the prediction of the radiomic signature: ECM-receptor interactions promote tumor cell migration and metastasis; the PI3K-Akt pathway regulates cell proliferation and survival; and adhesion plaques involved in ECM signaling serve as a central mechanism driving metastasis [85].

Beginning from the image prediction results, biological interpretation utilizes multi-omics technologies (e.g., transcriptome, metabolome, spatial genomics) to analyze the biological mechanisms between imaging features and clinical outcomes, as well as to excavate the molecular mechanisms behind them, so as to realize the closed-loop validation of the “Image-Organomics-Clinical” concept.

## Current challenges

Despite the remarkable success of radiomics and radiogenomics in the management of RCC, multiple challenges remain in achieving clinical translation. The core problem stems from variations in multiple parts of the imaging genomics workflow: random effects (e.g., patient positional differences, scanner noise) and systematic biases (e.g., cross-scanner differences, inter-observer segmentation inconsistencies) combine to result in a lack of stable radiomic features [86]. Furthermore, radiogenomics still faces the challenge of insufficient integration with other multi-omics approaches. Despite the increasing accumulation of genomics, transcriptomics, and proteomics data, the integration of radiomics with these multi-omics approaches is still at an early stage. In recent years, radiogenomics has effectively been supported by the rapid establishment of global molecular and imaging public databases, among which include the combination of TCGA and TCIA databases [87]. TCIA is a large-scale cancer medical image database containing CT, MRI, PET-CT, and pathology image data, and it also provides the corresponding omics data (e.g., genomics, transcriptomics, and proteomics data). In addition, the clinical translation of radiomics and radiogenomics is limited by the “black box” nature of deep learning algorithms, which is mainly due to the lack of explainable artificial intelligence (XAI), for which three types of nonvisual XAI methods have been proposed: case-based, textual, and auxiliary explanations. These measures can make models constructed from features carrying biological information highly interpretable [88]. Finally, most of the current results are based on small-sample retrospective studies, which can easily cause data bias and make the model less reproducible. Large-sample prospective studies are thus needed to further validate the reproducibility and stability of the model.

## Conclusions

The evolution from radiomics to radiogenomics has demonstrated transformative potential in the diagnosis and treatment of RCC. By leveraging high-throughput imaging features for accurate diagnosis and prediction, and correlating these with genetic mutations and TME characteristics, radiogenomics not only uncovers the biological mechanisms underlying imaging phenotypes but also guides clinical decision-making for optimizing targeted and immunotherapy in RCC. Despite the challenges posed by tumor heterogeneity, the integration of multi-omics technologies with deep learning promises to usher in an era of precision medicine in the diagnosis and treatment of RCC.

## Abbreviations

AUC: Area under the curve
CAF: Cancer-associated fibroblast
ccRCC: Clear cell renal cell carcinoma
chRCC: Chromophobe renal cell carcinoma
DFS: Disease-free survival
ECM: Extracellular matrix
mRCC: Metastatic renal cell carcinoma
OS: Overall survival
pRCC: Papillary renal cell carcinoma
RCC: Renal cell carcinoma
RF: Random Forest
TCGA: The Cancer Genome Atlas Program
TCIA: The Cancer Imaging Archive
TME: Tumor microenvironment
TNM: Tumor-node-metastasis
VB: Virtual biopsy
XAI: Explainable artificial intelligence
